# A monocentric analysis of the efficacy of extracellular cryoprotectants in unfrozen solutions for cleavage stage embryos

**DOI:** 10.1186/s12958-019-0519-2

**Published:** 2019-10-27

**Authors:** Francesco Capodanno, Jessica Daolio, Gaetano De Feo, Angela Falbo, Daria Morini, Alessia Nicoli, Luca Braglia, MariaTeresa Villani, Giovanni B. La Sala, Lodovico Parmegiani, Lorenzo Aguzzoli

**Affiliations:** 1 Center of Reproductive Medicine “P. Bertocchi”, Azienda Unità Sanitaria Locale – IRCCS di Reggio Emilia, Viale Risorgimento 80, 42123 Reggio Emilia, Italy; 2 Research and Statistics Infrastructure, Azienda Unità Sanitaria Locale - IRCCS di Reggio Emilia, Viale Risorgimento 80, 42123 Reggio Emilia, Italy; 3 Reproductive Medicine Unit, GynePro Medical Centers and NextClinics International, Via Tranquillo Cremona 8, 40137 Bologna, Italy

**Keywords:** ART, Cryopreservation, IVF, Slow-freezing, Vitrification, Warming

## Abstract

**Background:**

In the absence of international guidelines indicating the usage of vitrification rather than slow-freezing, the study aim was to analyze a large cohort of slow-frozen/thawed embryos to produce a rationale supporting the standardization of IVF cryopreservation policy.

**Methods:**

This retrospective analysis included 4779 cleavage stage embryos cryopreserved by slow-freezing/thawing from September 2009 to April 2017 at a single Center. Biological and clinical outcomes of three different commercial kits adopted sequentially, i.e. Vitrolife Cleave Kit® from Vitrolife (kit 1) vs. K-SICS-5000 Kit® and K-SITS-5000 Kit® from Cook Medical (kit 2) and Freeze/Thaw 1™ Kit® from Vitrolife (kit 3) were collected and compared in the light of cryoprotectants composition.

**Results:**

Kit 3 compared to kit 1 and kit 2 showed significantly (*P* < 0.001) higher embryo survival (79.9% vs. 75.6 and 68.1%, respectively) and frozen embryo replacement (91.5% vs. 86.5 and 83.3%, respectively) rates, and significantly (*P* < 0.001) lower blastomere degeneration rate (41.5% vs. 43.6 and 52.4%, respectively). No significant difference for clinical outcomes was observed among kits. Only a slight positive trend was observed for kit 3 vs. kit 1 and kit 2 on delivery rate per thawing cycle (7.12% vs. 4.19 and 4.51%, respectively; *P* < 0.058) and live birth rate (3.07% vs. 2.59 and 1.93%, respectively, *P* < 0.069). Thawing solutions of kit 3 were similar to those of any warming protocol.

**Conclusions:**

A defined concentration of extracellular cryoprotectants in thawing/warming solutions had a beneficial effect on the embryo cryosurvival rate. Results could provide the rationale for the adoption of a single standardized warming protocol.

## Background

Cryopreservation of human gametes and embryos permits to store the reproductive material in a viable state for undetermined periods of time. However, during embryo cryopreservation, the formation of intracellular ice can lead to cell damage and development arrest with a negative impact on cryosurvival ability [1–3]. To overcome these problems, in the last thirty-years there has been a continuous improvement and optimization of cryopreservation methods, protocols and solutions nowadays available on the market in ready-to-use kits that led cryopreservation to be applied in any in vitro fertilization (IVF) laboratory routinely. As consequence, to date, the efficacy and efficiency of reproductive cells cryopreservation contributes to the cumulative success rate of any IVF Center worldwide [2, 4–6]. In this sense, cryopreservation was proposed being an embryo treatment potentially able to improve the success rate in IVF couples rather than a strategy of embryo storage [7].

According to this definition, in 2016 the annual report from the Italian IVF National Registry reported that the 91.8% of the Italian IVF Centers carried out cryopreservation activities offering an overall 47% higher chance of pregnancy to each infertile couple [8]. Italian data are in line with those from the European Society of Human Reproduction and Embryology (ESHRE) as, between 2012 and 2013, the pregnancy rate of frozen embryo replacement (FER) cycles raised from 23.1 to 27% [9, 10].

In assisted reproductive laboratories human embryos at cleavage stage can be stored by two methods of cryopreservation: slow-freezing or vitrification [11]. Slow-freezing was the first cryopreservation method developed and leading to the first FER pregnancy [12]. It was used until the vitrification method has progressively replaced it at many IVF Centers worldwide [13–16] on the basis of several data reporting higher cryosurvival and blastulation rates [17–22]. To date, it is acknowledged that vitrification is superior to slow-freezing with a moderate quality of evidence regarding oocyte and embryo cryosurvival rates, but it is also known that the quality of evidence regarding clinical outcomes remains low by comparing the two methods [23].

Since international guidelines indicating the usage of vitrification rather than slow-freezing have still to be produced and cryopreservation protocols standardized, some IVF laboratories still adopt slow-freezing considering vitrification time consuming, operator-demanding and not as efficient as slow-freezing, especially for clinical outcomes related to embryos at cleavage stage [24, 25]. This could be in part due to different backgrounds regarding National Legislation on Reproductive Medicine regulating IVF policy in Countries.

In our realty we have cryopreserved embryos at cleavage stage by the method of slow-freezing/thawing for a period of time of almost a decade, during which three different ready-to-use kits were subsequentially introduced: K-SICS-5000®/K-SITS-5000® Kit (Cook Medical, USA), Freeze-Kit 1™/Thaw-Kit 1™ (Vitrolife, Sweden) and FreezeKit™ Cleave®/ThawKit™ Cleave® (new formulation, Vitrolife, Sweden). The adoption of kits was sebsequent as it followed the administrative rules belonging to our Hospital on consumable supplies.

Because of same changes in the clinical management of our IVF Center, the switch to the method of vitrification was proposed. As recent evidence has indicated that a typical laboratory could improve the embryo cryosurvival rate from almost 60% using slow-freezing to almost 78–100% using vitrification [23], we felt the obvious need to evaluate the efficacy and efficiency of our conventional cryopreservation activity prior to introduce the vitrification in our daily practice. As slow-freezing/thawing kits contained slight differences in their composition, we thus tested whether FreezeKit™/ThawKit™ Cleave® solutions accounted first for improved biological and clinical outcomes compared to kits K-SICS-5000/K-SITS-5000 and Freeze-Kit 1™/Thaw-Kit 1™, and, secondly, whether these were comparable to those reported for vitrification.

By this retrospective study on biological and clinical outcomes, we aimed to produce a rationale supporting the standardization of IVF cryopreservation policy focused on the composition of kits commercially available.

## Methods

### Design

This was a retrospective observational study collecting data on slow-frozen/thawed embryos from a cohort of infertile patients treated at the Center of Reproductive Medicine “P. Bertocchi” at the “*S. Maria Nuova*” hospital, AUSL – IRCCS in Reggio Emilia from September 2009 until April 2017 who gave their written consent for embryo cryopreservation. The study was approved by the local Ethical Committee.

### Population

In this study, inclusion criteria were confined to embryos for which both procedures of slow-freezing and thawing were performed using the same kit, and to thawing-survived embryos transferred in the contest of FER cycles.

Exclusion criteria were limited to embryos obtained from infertile couples in which the presence of severe male infertility factor (azoospermia), genetic disorders (i.e. cystic fibrosis or abnormal karyotype) in at least one partner, history and/or diagnosis of pelvic diseases (such as uterine malformations, endometriosis and/or pelvic inflammatory disease) and/or mayor medical conditions (such as diabetes mellitus, thyroid diseases, autoimmunity diseases, etc.) was assessed.

### Protocol

Controlled ovarian hyperstimulation was achieved using individualized protocols of gonadotropins, recombinant follicle stimulating hormone (FSH) or highly purified human menopausal gonadotropin in short or long gonadotropin-releasing hormone agonist down-regulated cycles. The criteria to start gonadotropin administration were serum estradiol (E2) concentration < 50 pg/ml and absence of follicle(s) with diameter > 10 mm. The ovarian response was monitored by use of serial transvaginal ultrasonographies and serum E2. In presence of at least 3 leading follicles with a mean diameter ≥ 18 mm, ovulation was triggered 24 h after the last gonadotropin injection using the human or recombinant chorionic gonadotropin (CG) administration.

Oocyte retrieval was performed by ultrasound-guided transvaginal aspiration 34–36 h after the triggering of ovulation. The luteal phase was supported by intravaginal progesterone. Semen samples were collected by masturbation after 3–5 days of abstinence. The preparation for conventional IVF or intracytoplasmic sperm injection (ICSI) was performed following the World Health Organization (WHO) standard protocol [26]. The absence of a follicular response after 35 days of treatment or a serum E2 value > 4000 pg/ml and/or > 20 follicles with a mean diameter > 10 mm during controlled ovarian stimulation was considered as indications for abandoning the cycle (cancelled cycle).

In the study period, embryos were scored according to classic parameters until December 2014 after which the Istanbul Consensus Workshop parameters were applied [27]. All the embryos evaluated in the first period were revisited and subsequently re-scored according to the newly international criteria [27]. Operators assessing embryo scoring acquired a documented training and experience according to requirements of the Italian State Regions Conference of March 15th, 2012.

Embryo scoring was performed on the day of embryo transfer and eligible supernumerary embryos were cryopreserved by slow-freezing. On the same day, embryos with an evident development arrest were considered not eligible either for transfer or for cryopreservation. Slow-freezing and thawing protocols were subsequently applied as detailed below.

Luteal phase support began on the 1st or 2nd day of a spontaneous or progesterone (P)-induced menstrual cycle according to a standardized protocol of our Centre. An oral administration of E2 valerate (Progynova; Shering, Milan, Italy) was given at a dose of 2 mg twice daily. Endometrial monitoring was performed by serial ultrasound assessments of endometrial thickness beginning from day 12. Intravaginal micronized P (Prometrium; Rottapharm, Milan, Italy) was initiated (200 mg twice daily) at 8–12 mm endometrial thickness. Embryo transfer was performed 3 days after beginning P therapy.

Medical treatment was continued until serum b-human chorionic gonadotropin (hCG) testing dosing 14th day after embryo transfer. In case of a positive b-hCG test a second assay was performed 48 h later. A transvaginal scan was performed 4 weeks after the second b-hCG assay to confirm the presence of an intrauterine gestational sac. In case of a negative b-hCG assay or confirmation of ongoing pregnancy, treatment was discontinued as soon as possible or after the 8th week of gestation, respectively.

#### Slow-freezing and thawing methods

According to the Italian Law 40/2004, supernumerary evolutionary embryos at cleavage stage were considered eligible for cryopreservation (2004) and slow-frozen on the day of embryo transfer. Independently on the kit in usage, the slow-freezing method was based on increasing concentrations of extracellular and intracellular cryoprotectant agents (CPAs) and on a slow stepwise freezing performed by an automated Planer Kryo 10 series III biological freezer (Planer Kryo 10/1,7 GB Planer, Plc, Sunbury-on-Thames, UK) as reported elsewhere [12, 28], whereas thawing method was performed by a rapid protocol based on a stepwise lowering of CPAs. Freezing/Thawing solutions and protocols were used and applied according to each specific manufacturer’s instructions detailed in Table 1 without any different operative change. The storage was performed using 0.25 ml Crystal CBS straws from 2009 until 2015 and 0.3 ml High Security CBS straws from 2015 up to now, both from CryoBioSystem®.

**Table 1:** Description of slow-freezing/thawing protocols used in the study period, including formulations and methods

	Kit 1 provided by Cook Medical (USA)	Kit 2 provided by Vitrolife (Sweden)	Kit 3 provided by Vitrolife (Sweden)
Freezing solutions	FS1: Cryo-PBS without CPAs FS2: Cryo-PBS with 1.5 M 1.2-propanediol FS3: Cryo-PBS with 1.5 M 1.2-propanediol and 0.1 M sucrose	FS1: Cryo-PBS without CPAs FS2: Cryo-PBS with 1.5 M 1.2-propanediol FS3: Cryo-PBS with 1.5 M 1.2-propanediol and 0.1 M sucrose	FS1: Cryo-PBS without CPAs FS2: Cryo-PBS with 1 M 1.2-propanediol and 0.5 M sucrose
Freezing method	Step 1: Equilibrate S1 for 10′ at 37° in air. and of S2 and S3 for 10′ at RT Step 2: Embryo incubation in S1 for 10′ at RT Step 3: Embryo incubation in S2 for 10′ at RT Step 4: Embryo incubation in S3 up to load in straws Step 5: Embryo loading and straws sealing Step 6: Loading of straws into the automatic freezer	Step 1: Equilibration of S1. S2 and S3 for 15′ at RT Step 2: Embryo incubation in S1 for 5′ at RT Step 3: Embryo incubation in S2 for 5′ at RT Step 4: Embryo incubation in S3 up to load in straws Step 5: Embryo loading and straws sealing Step 6: Loading of straws into the automatic freezer	Step 1: Equilibration of S1 and S2 for 15′ at RT Step 2: Embryo incubation in S1 for 10′ at RT Step 3/4: Embryo incubation in S2 and within 10′ embryo loading and straws sealing Step 5: Loading of straws into the automatic freezer
Automatic freezing program	Start temperature at + 20 °C; Lower to −7 °C at 3 °C per minute; Soak for 10′; Manual seeding of straws; Hold for 10′; − 7 °C to − 30 °C at 0.3 °C per minute; − 30 °C to − 150 °C at 50 °C per minute; Plunge into liquid nitrogen and storage.	Start temperature at + 20 °C; Lower to − 7 °C at 0.2 °C per minute; Manual seeding of straws; Hold for 7′; − 7 °C to − 30 °C at 0.3 °C per minute; − 30 °C to − 150 °C at 50 °C per minute; Plunge into liquid nitrogen and storage.	Start temperature at + 20 °C; Lower to − 6 °C at 0.2 °C per minute; Manual seeding of straws; Hold for 10′; − 6 °C to − 30 °C at 0.3 °C per minute; − 30 °C to − 150 °C at 50 °C per minute.
Thawing solutions	TS1: Cryo-PBS with 1.0 M 1.2-propanediol and 0.2 M sucrose TS2: Cryo-PBS with 0.5 M 1.2-propanediol and 0.2 M sucrose TS3: Cryo-PBS with 0.2 M sucrose TS4: Cryo-PBS without CPAs	TS1: Cryo-PBS with 1 M 1.2-propanediol and 0.2 M sucrose TS2: Cryo-PBS with 0.5 M 1.2-propanediol and 0.2 M sucrose TS3: Cyo-PBS with 0.2 M sucrose TS4: Cryo-PBS without CPAs	TS1: Cryo-PBS with 1 M sucrose TS2: Cryo-PBS with 0.5 M sucrose TS3: Cryo-PBS without CPAs
Thawing method	Step 1: Equilibrate TS1. TS2. TS3 and TS4 for 10′ at 20 °C Step 2: Thaw straws in air for 40″ and then in a water bath at 30 °C for 30″ Step 3: Cut the end of a straw. Fit syringe. Cut the other end of the straw and expel contents into a petri dish Step 4: Embryos incubation in TS1 for 5′ Step 5: Embryos incubation in TS2 for 5′ Step 6: Embryos incubation in TS3 for 5′ Step 7: Embryos incubation in TS4 for 5′ at RT and then for 5′ at 37 °C Step 8: Transfer embryos to equilibrated Cleavage Medium and incubate until transfer	Step 1: Equilibrate TS1. TS2. TS3 and TS4 for 10′ at 20 °C Step 2: Thaw straws in air for 30″ and then in a water bath at 30 °C for 30″ Step 3: Cut the end of a straw. Fit syringe. Cut the other end of the straw and expel contents into a petri dish Step 4: Embryos incubation in TS1 for 5′ Step 5: Embryos incubation in TS2 for 5′ Step 6: Embryos incubation in TS3 for 10′ Step 7: Embryos incubation in TS4 for 6′ at RT and then for 4′ at 37 °C Step 8: Transfer embryos to equilibrated Culture medium and incubate until transfer	Step 1: Equilibrate TS1. TS and TS3 for 10′ at RT Step 2: Thaw straws in air for 30″ and then in a water bath at 30 °C for 45″ Step 3: Cut the end of a straw. Fit syringe. Cut the other end of the straw and expel contents into a petri dish Step 4: Embryos incubation in TS1 for 5′ Step 5: Embryos incubation in TS2 for 5′ Step 6: Embryos incubation in TS3 for 5′ Step 7: Transfer embryos to equilibrated Culture medium and incubate until transfer

*FS1* Freezing solution number 1, *FS2* Freezing solution number 2, *FS3* Freezing solution number 3, *PBS* Phosphate-buffered saline, *CPAs* Cryoprotectants agents, *RT* Room temperature, *TS1* Thawing solution number 1, *TS2* Thawing solution number 2, *TS3* Thawing solution number 3, *TS4* Thawing solution number 4

From September 2009 to December 2010, we used Cryopreservation Kit K-SICS-5000® and Thawing Kit K-SITS-5000® from Cook Medical (USA) (kit 1); from January 2011 to April 2015, we used Freeze-Kit® 1™ and Thaw-Kit® 1™ from Vitrolife (Sweden) (kit 2); from May 2015 to April 2017, we used FreezeKit™ Cleave® and ThawKit™ Cleave® that was a new Vitrolife formulation (Sweden) (kit 3).

Embryos frozen with solutions of kit 1 were incubated in Cryo Dulbecco’s phosphate-buffered saline (PBS) without CPAs for 10 min at room temperature and then transferred in Cryo-PBS containing 1.5 M 1,2-propanediol (PROH – an intracellular CPA) for 10 min at room temperature. Embryos were finally exposed to a third solution of Cryo-PBS containing 1.5 M PROH plus 0.1 M sucrose (an extracellular CPA) to be loaded into straws. The straws were sealed and placed into the automated freezer, whose programmed cooling curve is reported in Table 1, before being plunged into liquid nitrogen at − 196 °C for long-term storage. During the thawing procedure, straws were removed from nitrogen and, after 40″ at room temperature, plunged in a water bath at 30 °C for 30″. Embryos were released into the first thawing solution with 1 M PROH and 0.2 M sucrose for 5′ and subsequently incubated for the same time in the second, third and fourth thawing solutions respectively containing 0.5 M PROH and 0.2 M sucrose, 0.2 M sucrose and no CPAs. Finally, embryos were transferred into the equilibrated Sydney IVF Cleavage Medium® (Cook Medical, USA).

Embryos frozen with solutions of kit 2 were incubated in Cryo-PBS without CPAs for 5 min at room temperature and then transferred in Cryo-PBS containing 1.5 M PROH for 5 min at room temperature. Embryos were finally exposed to a third solution of Cryo-PBS containing 1.5 M PROH plus 0.1 M sucrose to be loaded into straws. The straws were sealed and placed into the automated freezer, whose programmed cooling curve is reported in Table 1, before being plunged into liquid nitrogen. Thawing procedure and solutions of kit n.2 were the same of kit n.1 previously described, but embryos were exposed for 10 and 6 min at the third and fourth solution respectively and incubated for 4 min at 37 °C before transferred into the equilibrated Sydney IVF Cleavage Medium® (Cook Medical, USA).

Embryos frozen with solutions of kit 3 were incubated in a first Cryo-PBS solution without CPAs for 10 min at room temperature and then in a second Cryo-PBS solution containing 1 M PROH and 0.5 M sucrose; embryos were then loaded into High Security straws within 10 min. The straws were sealed and placed into the automated freezer, whose programmed cooling curve is reported in Table 1, before being plunged into liquid nitrogen. Thawing procedure and solutions of kit 3 excluded one step and containing only decreasing concentrations of sucrose as extracellular cryoprotectant. However, the procedure was similar to those previously reported. Embryos were exposed in air for 30″ and then in a water bath at 30 °C for 45″, incubated sequentially in the first, second and third thawing solution for 5′ each and then transferred into equilibrated Embryo Glue® transfer medium (Vitrolife, Sweden). Of note, all solutions of kit 3 contained a different base medium composition with amino acids for supporting embryo viability, MOPS buffer for physiological pH maintenance and hyaluronan to support embryo survival after cryopreservation.

All thawed embryos were checked under an inverted microscope throughout Hoffman modulation contrast by a magnification of 400X (TE 2000 U, Nikon Corp., Tokyo, Japan) to assess the morphological evaluation and survival. Partially or full-survived thawed embryos were allocated to FER if they had at least one intact blastomere or more surviving cells. These last were incubated at 37 °C with 6% CO_2_ at least one hour before FER was performed. Frozen/thawed embryos presenting full blastomere degeneration were discarded.

### Endpoints and data collection

To compare the biological and clinical efficiency between kits 1, 2 and 3, biological outcomes included were the embryo survival rate, the blastomere degeneration rate and FER rate, and clinical outcomes included were the implantation rate, the clinical pregnancy rate, the abortion rate, the delivery rate and the live birth rate.

The embryo survival rate was calculated as the percentage of transferred embryos among thawed embryos, the degeneration rate of blastomeres was calculated as the percentage of surviving thawed blastomeres among frozen blastomeres and the FER rate was calculated as the percentage of FER events among thawing cycles.

The implantation rate was calculated as the percentage of gestational sacs among transferred embryos and the clinical pregnancy rate was calculated per thawing cycle and per FER. Of note, only clinical pregnancies certified by the presence of at least one gestational sac at ultrasound were considered for calculation of implantation rate. Abortion rate was calculated as the percentage of events among clinical pregnancies, the delivery rate was calculated as the percentage of deliveries among total pregnancies and per thawing cycle and the live birth rate was calculated as the percentage of live births among thawed embryos.

### Statistical analysis

In absence of a-priori hypothesis and given the exploratory nature of the study, no formal sample size calculation was performed. Between-kit differences in ratios were analyzed using χ2-tests or Fisher’s exact test when at least one cell expected numerosity under rows columns independence assumption was below 5. Statistical analysis was performed by using R 3.4.4 software [29] and *P*-values less than 0.05 were considered being significant.

## Results

A total number of 4779 embryos at cleavage stage was slow-frozen/thawed accounting for 2024 thawing cycles performed. From September 2009 to December 2010, a total of 618 cleavage stage embryos were obtained from 288 patients (mean age 36.1 ± 3.7 years) and slow-frozen/thawed with kit 1; from January 2011 to April 2015, a total of 2856 cleavage stage embryos were obtained from 916 patients (mean age 35.8 ± 3.8 years) and slow-frozen/thawed with kit 2; from May 2015 to April 2017, a total of 518 cleavage stage embryos were obtained from 418 patients (mean age 35.7 ± 4.0 years) and slow-frozen/thawed with kit 3.

Clinical and biological outcomes were showed in Table 2. Age and number of transferred embryos (per patient) were compared between groups via ANOVA. The age of patients was similar between groups (*P* = 0.329). The number of transferred embryos per patient related to kit 3 was significantly higher compared to kit 1 and 2 (*P* < 0.001). Embryo survival rate of kit 3 was significantly higher compared to kits 1 and 2 (*P* < 0.001). Significantly lower blastomere degeneration rate was observed in the same group compared to kits 1 and 2 (*P* < 0.001). FER rate related to kit 3 was significantly higher compared to kits 1 and 2 (*P* < 0.001).

**Table 2:** Clinical and biological outcomes

	Kit 1	Kit 2	Kit 3	*P* value
N. of patients	288	966	418	
Age of female partners (mean ± SD)	36.06 ± 3.73	35.76 ± 3.76	35.65 ± 4.04	0.329
Transferred embryos per patient (mean ± SD)	1.54 ± 1.15	1.77 ± 1.58	2.27 ± 1.80	< 0.001*
Survival rate of thawed embryos (%)	75.57	68.14	79.92	< 0.001*
Degeneration rate of blastomeres (%)	43.58	52.38	41.48	< 0.001*
FER rate (%)	86.45	83.38	91.54	< 0.001*
Implantation rate (%)	5.14	4.83	5.08	0.936
Clinical pregnancy rate per thawing cycles (%)	6.45	8.27	9.23	0.370
Clinical pregnancy rate per FER (%)	7.46	9.92	10.08	0.436
Abortion rate (%)	35	24.24	20.83	0.459
Delivery rate per total pregnancies (%)	44.83	37.76	49.33	0.246
Delivery rate per thawing cycle (%)	4.19	4.51	7.12	0.058
Live birth rate (%)	2.59	1.93	3.07	0.069

*Significant (*P* < 0.05) difference between kit 1, kit 2 and kit 3. *FER* Frozen Embryo Replacement, *SD* Standard deviation

Implantation rate, clinical pregnancy rate per thawing cycles and per FER, abortion rate, delivery rate per total pregnancy and per thawing cycles as well as live birth rate were all no significant different between all the three study groups. However, a slight positive trend in favor of kit 3 was observed regarding the delivery rate per thawing cycle and the live birth rate (*P* = 0.058 and 0.069, respectively).

Previously unplanned multivariate logistic analyses confirmed non statistical significant effect of group on clinical pregnancies accounting for number of transferred embrios (results not shown).

## Discussion

Embryo cryopreservation has become an established procedure in the field of reproductive medicine [12, 30–32] accounting for a cumulative 28% of delivery rate following IVF programs at least in Europe [4, 10, 33]. Slow-freezing was the first technique of cryopreservation employed in IVF laboratories, but, in the last years, it has been progressively replaced by the method of vitrification of the basis of a quantity of literature recently reviewed reporting higher cryosurvival rate in both cleavage- and blastocyst-stage embyos [23]. However, International guidelines have still to be produced prompting IVF laboratories to optimize cryopreservation protocols in order to achieve the best clinical management of reproductive cells. In this perspective, some studies tested the possibility of using a universal medium to unfreeze any cell independently on the freezing protocol adopted to simplify the management of reproductive cells between IVF Centers [34–37].

In our IVF laboratory practice, embryo cryopreservation policy has been always based on the method of slow-freezing. In light on the most recent quality of evidence [23], before abandoning the conventional protocol, we critically evaluated results on biological and clinical outcomes from almost a decade of slow-freezing activity by comparing three different ready-to-use commercial kits as a prelude to the use of vitrification.

The adoption of kit 3 had a positive impact on our biological results as we observed higher embryo survival rate and lower blastomere degeneration rate that, combined, allowed us to perform more than 90% of FER cycles. The number of embryos to thaw and transfer did not vary in the study period. Nonetheless, kit 3 allowed us to transfer more thawed embryos per patient compared to kits 1 and 2. In our opinion, this was due to the higher cryosurvival rate of kit 3 that favored the transfer of more cryosurvived embryos per patient.

The analysis of data also revealed that kit 3 protocol was more efficient than kits 1 and 2 representing the best slow-freezing method applied to cleavage stage embryos employed in our IVF laboratory so far. The observed improvement of kit 3 on embryo cryosurvival rate was in accordance with that reported in a previous study in which the efficacy of the same kit was compared to another commercial composition of solutions for slow-freezing [25]. Interestingly, in our realty, the cryosurvival rate of kit 3 was improved to almost 80% as recently suggested to be expected from a typical laboratory using vitrification [23].

During the study period, kits 1, 2 and 3 were adopted sequentially in our IVF laboratory without changes applied to our cryopreservation policy and consent for patients. Cryopreservation protocol was not changed, if not specified and required by the manifacturer’s instructions. We considered these changes as intrinsic variables of each kit that mirror a clinical and technical realty constantly growing.

Focusing on kits composition, kit 1 was a formulation by Cook Medical (USA) made by three freezing and four thawing solutions, whereas kits 2 and 3 were two different formulations by Vitrolife (Sweden) providing three and two freezing combined respectively to four and three thawing solutions (Table 1). Kit 3 protocol excluded one step during freezing and thawing which made the procedure more time-efficient.

All slow-freezing solutions contained PBS medium supplemented with PROH and sucrose as permeating and non-permeating CPAs at a maximum concentration of 1–1,5 mol/l and 0,1–0,5 mol/l respectively; differently, the cryoprotectants in the freezing solutions of kit 3 were the same as in kits 1 and 2, but the concentrations of sucrose were higher. On the other hand, thawing solutions of kits 1 and 2 contained both permeating and non-permeating CPAs, PROH and sucrose, but those of kit 3 contained only sucrose. Thawing solutions of kit 3 are quite similar to those used during rapid warming in any vitrification protocol. Interestingly, the extracellular concentration of sucrose as CPA in the first warming solution of any vitrification protocol together with high warming rates are the two key performance features of vitrification technique that optimize cell survival by preventing recrystallization and cell lysis [38, 39]. In this perspective, kit 3 fits better both features of vitrification compared to kits 1 and 2, resulting in accordance with the rationale of a previous study demonstrating that, irrespective of the freezing protocol, a rapid warming procedure by stepwise dilutions of extracellular cryoprotectants only, as sucrose, may be adopted for higher survival rate of both slow-frozen and vitrified reproductive cells [34]. The use of only extracellular cryoprotectant (sucrose or threalose) in the thawing solution seems to be the best option for any frozen cell or tissue, as confirmed by the studies on clinical efficiency of Parmegiani’s Universal Warming [40, 41].

If, on one side, kit 3 performed as well as vitrication considering the improved cryosurvival rate, on the other side, it supported that vitrification applied to cleavage-stage embryos is not superior to slow-freezing when considering clinical outcomes, such as the clinical pregnancy rate and the live birth rates per cycle and transfer, as recently reviewed by Rienzi et al. [23]. Infact, the current study also compared clinical outcomes between kits, failing to demonstrate significant improvements regarding the implantation rate, the clinical pregnancy rate, the abortion rate, the delivery rate and the live birth rate. In our opinion, it may be explained by two main reasons: the female mean age at freezing was similar between groups suggesting that characteristics of patients treated and candidated to embryo cryopreservation did not change in the study period, and the criteria for the embryo evaluation by the embryologists as well as for the selection of embryo cryopreservation had not been modified at out Center, limiting the bias related the quality of thawing-survived embryos transferred between groups in the contest of FER cycles. However, the absence of a simple size calculation, the exploratory nature of the study and the differences in the length of periods of kits usage may constitute biases affecting our results.

## Conclusions

The adoption of kit 3 led us to optimize our cryopreservation policy by applying the method of slow-freezing to cleavage stage embryos. The similarity in the composition of the extracellular cryoprotectants between thawing solutions of kit 3 and conventional rapid warming solutions likely allowed to achieve the embryo cryosurvival rate expected by using vitrification, i.e. 78–100%. Given that, the current study definitively supported the shift to vitrification in our IVF laboratory to maximize the cumulative efficiency of FER cycles and paved the way for the adoption of a single standardized warming protocol in the next future. This last will also simply the management of reproductive cells between IVF Centers.

## Data Availability

The datasets used and/or analysed during the current study are available from the corresponding author on reasonable request.

## Abbreviations

CG: Chorionic Gonadotropin
CPAs: Cryoprotectant Agents
E2: Estradiol
ESHRE: European Society of Human Reproduction and Embriology
FER: Frozen Embryo Replacement
FSH: Follicle Stimulating Hormone
hCG: human Chorionic Gonadotropin
ICSI: Intracytoplasmic Sperm Injection
IVF: In Vitro Fertilization
P: Progesteron
PBS: Phosphate-Buffered Saline
PROH: 1,2-Propanediol
WHO: World Health Organization
